# Morphological Stasis in Time? A *Triatoma brasiliensis brasiliensis* Study Using Geometric Morphometrics in the Long Run

**DOI:** 10.3390/ani12111362

**Published:** 2022-05-26

**Authors:** Letícia Paschoaletto, Carolina Dale, Vanessa Lima-Neiva, Ana Laura Carbajal-de-la-Fuente, Jader de Oliveira, Hugo A. Benítez, Jane Costa

**Affiliations:** 1 Laboratório de Biodiversidade Entomológica, Instituto Oswaldo Cruz, Fiocruz, Rio de Janeiro 21040-361, Brazil; leticia.paschoaletto@gmail.com (L.P.); dale@ioc.fiocruz.br (C.D.); neivalv@gmail.com (V.L.-N.); 2 Programa de Pós-Graduação em Formação em Ciências para Professores, Universidade Federal do Rio de Janeiro, Rio de Janeiro 21941-617, Brazil; 3 Consejo Nacional de Investigaciones Científicas y Técnicas (CONICET), Buenos Aires 1063, Argentina; analaura.carbajal@gmail.com; 4 Centro Nacional de Diagnóstico e Investigación en Endemo-Epidemias (CeNDIE), Administración Nacional de Laboratorios e Institutos de Salud (ANLIS)—Ministerio de Salud y Desarrollo Social de la Nación, Buenos Aires 1063, Argentina; 5 Laboratório de Entomologia em Saúde Pública, Departamento de Epidemiologia, Faculdade de Saúde Pública, Universidade de São Paulo, São Paulo 05508-070, Brazil; jdr.oliveira@hotmail.com; 6 Laboratorio de Ecología y Morfometría Evolutiva, Centro de Investigación de Estudios Avanzados del Maule, Universidad Católica del Maule, Talca 3466706, Chile; 7 Centro de Investigación en Recursos Naturales y Sustentabilidad (CIRENYS), Universidad Bernardo O’Higgins, Avenida Viel 1497, Santiago 8370993, Chile

**Keywords:** morphology, Chagas disease, vectors, wing shape

## Abstract

**Simple Summary:**

Triatomines are vector insects capable of transmitting the protozoan that causes Chagas disease, thus representing a health risk in several countries, especially in Central and South America. *Triatoma brasiliensis brasiliensis*, the main triatomine vector in northeastern Brazil, needs frequent monitoring as it is able to colonize various natural and artificial ecotopes as well as to infest domiciles. This research uses geometric morphometrics as a tool to evaluate changes in the morphology and analyze a large temporal dataset of 102 years of collections of *T*. *b. brasiliensis.* Finding novelty results which indicate a morphological stasis in the morphology.

**Abstract:**

*Triatoma brasiliensis brasiliensis* Neiva, 1911 is one of the most important vectors of Chagas disease in the Brazilian semiarid regions in the north-east. The risk imposed by *T. b. brasiliensis* to the human populations, due to frequent invasions and/or colonization of the domiciles, demands constant monitoring and control actions as well as an understanding of its evolutionary process. In this context, the following research studies the pattern of shape adaptation over time using a large dataset from 102 years of specimen collections in order to identify the morphological plasticity of this vector in Brazil. This dataset was analyzed using geometric morphometrics tools and the timescale was divided into eight different groups, containing specimens from 1912 to 2014. Geometric morphometrics analysis showed an interesting morphological stasis in the wing shape of *T. b. brasiliensis*, which allowed us to understand the high capacity of adaptation to changes in climate condition through time, and the invasive status which *Triatoma* species have around the world. Moreover, these results showed novel findings as an interesting phenotypic pattern, with no modifications in more than 100 years, leading us to understand the shape evolution in Triatominae as a vector species of diseases.

## 1. Introduction

Chagas disease (CD) is caused by the protozoan *Trypanossoma cruzi* (Chagas, 1909) (Trypanosomatida, Trypanosomatidae), and is transmitted to humans mainly through hematophagous insects of the Triatominae subfamily. These insects suck human blood and defecate during or shortly after the blood repasture, leaving infective forms of *T. cruzi* that enter the host through skin wounds or mucosal membranes. Besides this form of vectorial transmission, other mechanisms are possible, such as blood transfusion, organ donation, oral ingestion (of food contaminated with the parasite), vertical transmission (mother to child during pregnancy), and as a result of laboratory accidents [1]. In this context, Chagas disease or American Trypanosomiasis remains as one of the most important and yet neglected diseases in the world [2,3].

Currently, there are more than 155 triatomine species, all considered to be potential vectors of *T. cruzi* [4,5]. In Brazil, a diverse fauna of triatomines is recorded, many of them composing species complexes. *Triatoma brasiliensis* complex, for example, is a monophyletic group comprising two subspecies and six species: *Triatoma brasiliensis brasiliensis* Neiva, 1911, *Triatoma petrocchiae* Pinto & Barreto 1925, *Triatoma melanica* Neiva & Lent 1941, *Triatoma brasiliensis macromelasoma* Galvão, 1956, *Triatoma bahiensis* Sherlock & Serafim, 1967, *Triatoma lenti* Sherlock & Serafim, 1967, *Triatoma sherlocki* Papa, Jurberg, Carcavallo, Cerqueira & Barata, 2002, and *Triatoma juazeirensis* Costa & Felix, 2007. Occurring in 12 Brazilian states, this complex is quite spread out in the country and colonizes mainly the *caatinga* and *cerrado* biomes [6,7,8,9]. The cerrado biome has strong climatic seasonality, and is characterized by alternating between rainy periods, during spring and summer, and dry periods, in autumn and winter. Its vegetation comprises forest, savanna and grassland formations [10]. The caatinga biome, on the other hand, is composed of low trees and shrubs with xerophytic characteristics, in addition to presenting a rainfall regime with great water deficit throughout the year [11].

*Triatoma brasiliensis brasiliensis* is one of the most important vectors of Chagas disease in the semiarid regions in Brazil [6,7,12] and occurs in six states: Ceará, Maranhão, Paraíba, Pernamb uco, Piauí, and Rio Grande do Norte. This species displays eclectic feeding behavior and can inhabit different ecotopes, besides presenting one of the highest rates of *T. cruzi* infection and high rates of intradomiciliary infestation [7,8,13,14,15,16,17,18]. The epidemiologic importance of *T. b. brasiliensis* has motivated a variety of studies, placing it as one of the most studied species of triatomines. The substantial amount of information available on its morphological, behavioral, biological, and eco-epidemiologic aspects has been providing subsides to improve the strategies of Chagas disease control programs in endemic areas [7,8,12,18,19,20,21,22].

Geometric morphometrics (GM) have been applied to the study of triatomines for a wide variety of approaches showing resolution, precision, and convergence with molecular tools [22,23,24]. Dujardin et al. [25,26] used GM analyses to differentiate domestic and wild populations of *Triatoma infestans* from Bolivia and to detect, through changes in sexual dimorphism of *Rhodnius* and *Triatoma* species, the transition from wild habitats to artificial ones, respectively. Lunardi et al. [27] applied this technique to compare specimens of *Triatoma williami* fed with different food sources in the Amazon, Brazil. Later, different peridomestic habitats of *T. infestans* in Bolivia were analyzed by the power of GM [28,29]. For *T. b. brasiliensis*, several evolutionary aspects were explored by means of GM revealing, for the first time, the homoploidal hybrid speciation process in the triatomine group [30].

The risk posed by *T. b. brasiliensis* to the human populations, due to constant invasions and/or colonizations, emphasizes the necessity of studies on its morphological variability to evaluate possible morphological changes over time, which could also be related to eventual evolutionary trends. The objective of this study is to broaden the available knowledge of this vector by seeking population characteristics that could clarify whether there were phenotypic changes possibly correlated to its genetics over time. This piece of information is of utmost importance to the understanding of the evolutionary processes of *T. b. brasiliensis* as it will add relevant data for the monitoring of infestations and aspects of its vectorial potential. This will assist in providing strategic plans for the control measures.

## 2. Materials and Methods

### 2.1. Origin and Identification of T. b. brasiliensis Specimens

This study was conducted with specimens of *T. b. brasiliensis* deposited in two entomological collections: a) the Entomological Collection of Instituto Oswaldo Cruz (CEIOC) and b) the Collection of Triatomines of Instituto Oswaldo Cruz (CTIOC), both in Brazil.

The specimens used come from different localities in six states in northeastern Brazil. Identification of the specimens was confirmed according to Lent & Wygodzinsky [31]; Costa et al. [6] and Dale et al. [9].

A total of 111 specimens of *T. b. brasiliensis*, 46 males and 65 females, were analyzed. Of these, 40 were from CEIOC and came from field captures carried out in 1996 and 2002, in the states of Rio Grande do Norte and Paraíba, respectively. The remaining 71 were deposited in the CTIOC and were collected in different periods and states (Table 1). The insects were separated according to the year of capture and then grouped, which culminated in the formation of eight groups for the analysis, comprising specimens from 1912 to 2014 (Table 1).

Ten specimens (five males and five females) of T. infestans collected in the municipality of Santa Rosa, Rio Grande do Sul, Brazil, during 2014, were used as an external group, in the final analyses performed.

### 2.2. Wings Preparation

The left hemelytron of both sexes was used. Those hemelytra that did not show the defined number of anatomical reference points were excluded. All wings suitable for the analysis were classified according to sex, year of collection, and geographical origin, and were photographed using a Nikon Coolpix 990 camera (Tokyo, Japan).

### 2.3. Data Collection

Ten landmarks were selected and identified from digital photos of each wing:1. Intersection of Pcu and Pcu + first anal vein; 2. Intersection of Cu and Cu–postocubitus (Cu–Pcu); 3. Intersection of Cu and M–Cu; 4. Intersection of media and cubitus (M–Cu); 5. Bifurcation of the radius (R) and median (M) veins; 6. Membrane portion on radius vein; 7. First intersection of R + M and Pcu (postocubitus); 8 Second intersection of R + M and Pcu (postocubitus); 9. Intersection of M and extension of Cu–Pcu veins; 10. Intersection of Pcu and Cu. 

**Figure 1:**
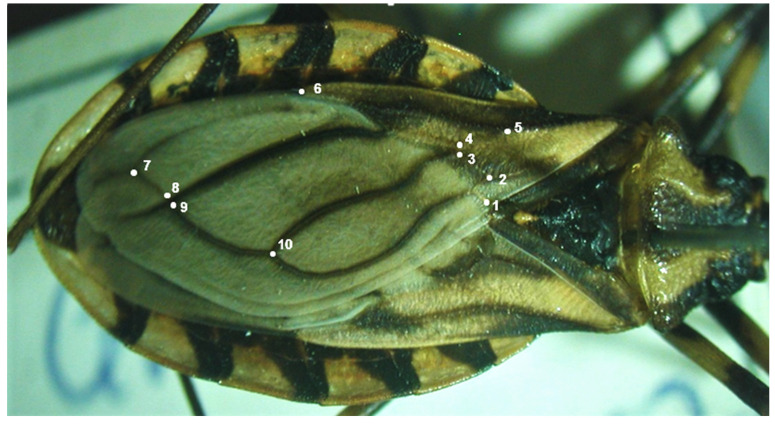
Image of the left wing of a male of *Triatoma brasiliensis brasiliensis* Neiva, 1911 showing the selected 10 landmarks: 1. Intersection of Pcu and Pcu (postocubitus) + first anal vein; 2. Intersection of Cu (cubitus) and Cu–postocubitus (Cu–Pcu); 3. Intersection of Cu and M–Cu; 4. Intersection of media and cubitus (M–Cu); 5. Bifurcation of the radius (R) and median (M) veins; 6. Membrane portion on radius vein; 7. First intersection of R + M and Pcu (postocubitus); 8 Second intersection of R + M and Pcu; 9. Intersection of M and extension of Cu–Pcu veins; 10. Intersection of Pcu and Cu.

These points were determined according to Oliveira et al. [23] (Figure 1). The landmarks were digitized by means of the software TPSDig2 v2.31 [32,33].

### 2.4. Geometric Morphometrics Analyses

The main method for GM analysis is the Procrustes superimposition, which removes mathematical information produced by size, position, and orientation [34,35]. This analysis was performed using TPSRelw v1.7 [36]. Additionally, the measurement error was analyzed and found to be negligible. Fruciano [37] indicates that measurement errors in GM can inflate the amount of variance and, since morphological analyses are based on the amount of “explained” relative to “residual” variance, the misposition of landmarks could influence the results.

TPSRelw shows two types of shape deformation: the first is the average shape, and the second one is obtained by the partial and relative wraps and denotes the shape changes of each individual. This process is also called principal component analysis, which represents the shape space of possible variation of every specimen [38,39].

The Lambda test of Wilks evaluated the multivariate differences between the groups [40]. To identify the variation among the groups, a canonical variate analysis (CVA) was performed using the shape data categorized by chronological order. With the results of the CVA, average distances were extracted and an unweighted pair group method with arithmetic mean (UPGMA) dendrogram was built to identify the relationships among the groups studied. All these analyses were performed using the software JMP (SAS Institute Inc., Cary, NC, USA version 3.2.2).

The centroid size measurements were used to reduce the size of the wings to a single variable [41] and the measurements and statistical significance among groups were tested by means of the analysis of variance (ANOVA), whereas the Tukey test was applied for the analysis between pairs.

## 3. Results

### 3.1. Morphometric Analysis

#### 3.1.1. Analysis of the Females Wings

**Figure 2:**
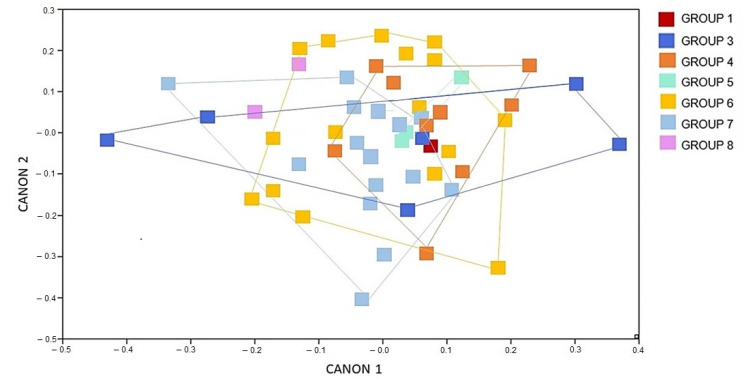
Canonical variate analysis showing the wing shape influence of the of *Triatoma brasiliensis brasiliensis* females from various states and years (1912–2014) Canon represents the canonical variate dimension.

The CVA of the female specimens of *T. b. brasiliensis* shows a big overlap among the groups studied, meaning that the left-wing shape is not largely different among chronological groups (Figure 2).

**Figure 3:**
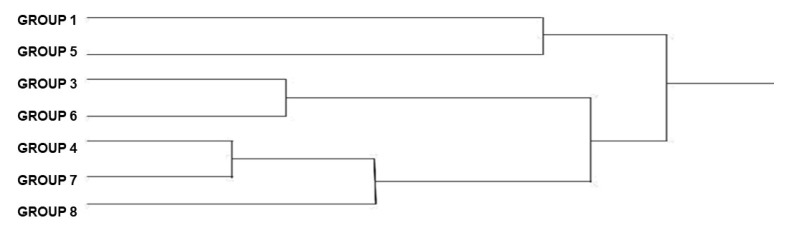
UPGMA dendrogram of the *Triatoma brasiliensis brasiliensis* females, from various states and years (1912–2014) showing the phylogenetic distances among the groups. Groups: 1, 3, 4, 5, 6, 7, 8.

The cluster analysis (Figure 3), considering the average of Mahalanobis distances among specimens, generated a dendrogram that shows all the overlapping groups, indicating that there has been no change over the years. A larger cluster that included more groups showed greater similarity in wing shape between groups.

The dendrogram (Figure 3), which shows the proximity among the populations, demonstrates that the specimens from group 8 are separated from the others, being the most differentiated group.

There is greater proximity between groups 1 and 5, 3 and 6, and between groups 4 and 7 (Figure 3).

#### 3.1.2. Analysis of the Males Wings

**Figure 4:**
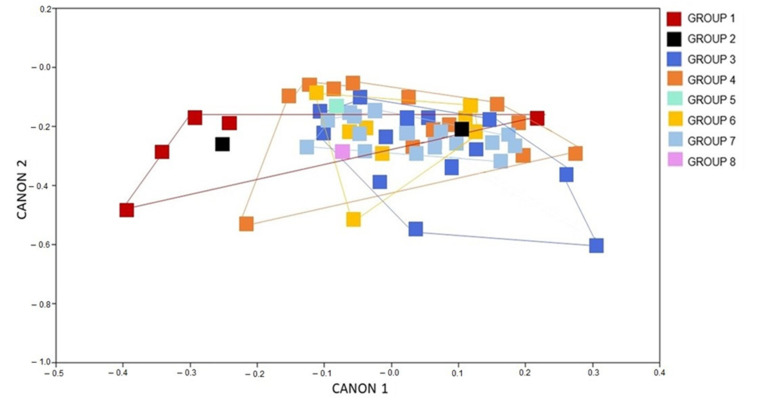
Canonical variate analysis showing the wing shape influence of *Triatoma brasiliensis brasiliensis males* from several states and years (1912–2014). Groups: 1, 2, 3, 4, 5, 6, 7, 8 Canon represents the canonical variate dimension.

The results observed in the CVA referring to the male populations corroborate the results obtained for females: an overlap of geometric shapes suggests that there was no phenotypic change determined by the chronological groups (Figure 4).

**Figure 5:**
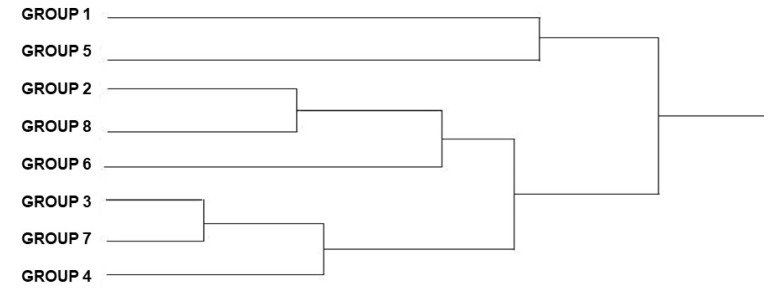
UPGMA Dendrogram of the *Triatoma brasiliensis brasiliensis* males, from various states and years (1912–2014), showing the phylogenetic distances between the groups.

The dendrogram (Figure 5) shows that there are more similarities among specimens in groups 1 and 5, 2 and 8, and 3 and 7.

#### 3.1.3. Analysis of the Males and Females Wing Shape

**Figure 6:**
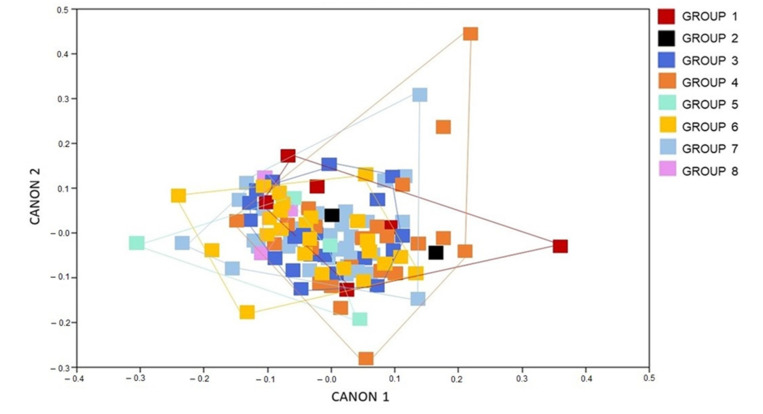
Canonical variate analysis showing the conformation influence in males and females of *Triatoma brasiliensis brasiliensis* from different states and years (1912–2014). among the groups Canon represents the canonical variate dimension.

A third analysis was carried out, now with male and female populations of *T. b. brasiliensis* together. The populations are structured in overlapping areas, suggesting that they remain unchanged in their phenotypes (Figure 6).

**Figure 7:**
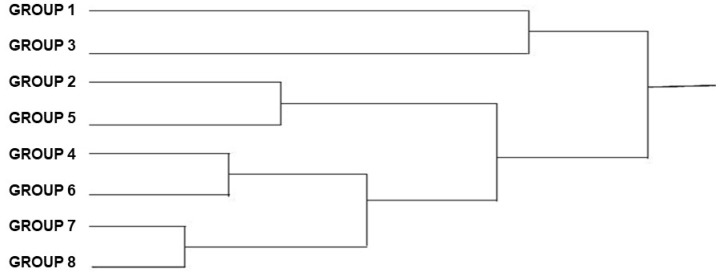
UPGMA dendrogram of the male and female populations of *Triatoma brasiliensis brasiliensis* from different states and years (1912–2014), showing the phylogenetic distances among the groups.

The dendrogram (Figure 7) showing the proximity among these groups determined a closer proximity for the groups 1 and 3, 2 and 5, 4 and 6, and between groups 7 and 8 (Figure 7).

**Figure 8:**
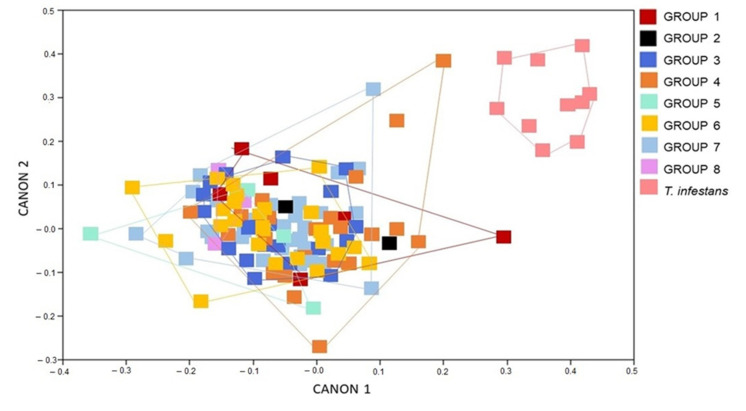
Canonical variate analysis showing the conformation influence in males and females of *Triatoma brasiliensis brasiliensis* from several states and years (1912–2014). Groups: 1, 2, 3, 4, 5, 6, 7, 8, and an external group *T. infestans* Canon represents the canonical variate dimension.

An additional analysis was carried out among all the groups studied and an external group, composed of 10 specimens of *T. infestans* collected in Rio Grande do Sul, to verify the possible level of morphological convergence of the *T. b. brasiliensis* groups (Figure 8). The result of the analysis ratifies the previous ones, in which all groups overlap.

The CVA below shows the external group (*T. infestans*) separated from the other groups of *T. b. brasiliensis*.

## 4. Discussion

The following research found that geometric morphometrics tools were not powerful enough to detect differences in wing morphology on a timescale of more than 100 years of *T. b. brasiliensis.* The analysis applied to the wing shape showed similar phenotypes around the whole set of landmarks for the eight groups of years. Morphological stasis founded in this data has been observed in highly invasive species such as *Drosophila* spp., in which there is an evolutionary trend to keep the shape morphology in order to keep their invasive behavior [42,43,44].

In the triatomine group, the GM analyses have been applied in a wide variety of approaches, allowing for the understanding of the speciation process and morphological differentiation. Gumiel et al. [45] used GM in taxonomic studies, resulting in the classification of *Triatoma melanosoma* as a junior synonym of *T. infestans*. Furthermore, in a study by Schachter-Broide et al. [46], GM was used to analyze *T. infestans* wings and, from the simultaneous analysis of both size and shape, it was possible to clarify the direction and elapsed time of the insect dispersion, establishing the relevance of this type of study to corelate morphological heterogeneities to the reinfestation patterns. Nattero et al. [47] concluded that the shape of the wings could be considered a reasonably good phenotypic marker, as it made it possible to distinguish the four species of the *sordida* subcomplex through GM analyses.

In the *T. brasiliensis* species complex, several applications of GM have revealed important aspects of its evolutionary history [30] and systematics [14,22]. Costa et al. [30] and Dujardin et al. [48] highlight the fact that triatomines can present a phenotype change due to environmental pressures, and this is a gradual process shown to parallel evolutionary relationships among closely related species. In the case of the members of the monophyletic group *T. brasiliensis* complex, it was suggested that the speciation process, also revealing morphological changes between *T. b. brasiliensis* and *T. melanica*, could have taken 5.2 million years, supposedly in the early Pliocene [49]. In this study, the analysis of the 111 *T. b. brasiliensis* specimens, from different states in the north-east of Brazil, from 1912 to 2014, showed no changes in the wing phenotype. A recent study based on molecular and morphological markers showed similar results in populations of *T. b. brasiliensis* from Rio Grande do Norte and Paraíba [22]. It is important to stress that the specimens here studied presented the typical color pattern of the *T. b. brasiliensis* according to previous descriptions in the literature [6,9,31].

Gurgel-Gonçalves et al. [50] combined GM and niche modeling to characterize two closely related species, *T. sordida* and *T. garciabesi*, obtaining satisfactory results in their studies. This approach emphasizes the power of GM to characterize morphologically cryptic species. Therefore, the separation of *T. infestans* as the outgroup from all the other *T. b. brasiliensis* groups corroborates previous studies in indicating that GM is an important tool for systematics and evolutionary relationships analyses and lends support in identifying the morphological stability of the *T. b. brasiliensis* over the last 100 years.

It is worth mentioning that the Northeast region is composed of four geographical areas: *Meio-norte* (mid-north), *Sertão* (hinterland), the semiarid *Agreste* region, and *Zona da Mata* (forest area). The *Sertão* is the largest, spreading over eight northeastern states—Piauí, Ceará, Rio Grande do Norte, Paraíba, Pernambuco, Alagoas, Bahia, and Sergipe. This vast area with a semi-arid climate is covered by a typical kind of vegetation known as *caatinga*. An ecological niche modeling was carried out to evaluate the climate and verify possible climatic environmental changes [7]. It was observed that this area presents climate stability without indication of possible changes for the next 50 years. This geographic–climatic stability could also be a factor influencing the morphological stability observed in *T. b. brasiliensis*.

A similar analysis has been performed with specimens of *T. infestans* with a time frame of over 70 years [51]. Regarding this species, which specifically occupied domiciliary ecotopes (in Brazil), where high climatic stability is also expected, no significant changes were observed in the different populations over the years. Although the dispersion history of *T. infestans* is quite distinct from that of *T. brasiliensis*, both species apparently occupy stable ecological spaces [7,52]. *Triatoma infestans* exclusively occupies the intradomicile in Brazil, whereas *T. b. brasiliensis* occupies several natural and artificial ecotopes. Both species are in different ecological spaces but in stable conditions of occupation.

In a recent study by Vilaseca et al. [29], GM was able to detect morphological variation of *T. infestans* specimens that inhabited two geographically distinct environments in Bolivia. This approach allowed for a better understanding of the biological adaptation of this species since the results showed the influence of different environmental factors on the size and shape of this species.

## 5. Conclusions

This research presents a novelty finding in which no differences were observed between multiple populations of the vector of Chagas disease, *T. b. brasiliensis*, for 102 years. Several reasons could explain this morphological stasis, nevertheless, the invasive force and the protozoan vector’s capacity to be spread could be explored in future studies. It is important to notice that according to the distributional potential analysis, no external forces have been favoring changes in this species morphology given the climatic and geographic stability in the main area infested by *T. b. brasiliensis*, in the north-east region of Brazil. In consonance with the GM results, *T. b. brasiliensis* is going to continue to be characterized by a specific and stable phenotype, which facilitates its identification by the technicians of the public health services responsible for the monitoring activities in the infested areas in Brazil. *Triatoma b. brasiliensis* is a native species, occurs in natural and artificial ecotopes and, consequently, cannot be eliminated by the control programs. It also presents a broad geographic distribution and is considered a relevant *T. cruzi* vector; therefore, *T. b. brasiliensis* is going to continuously impose a risk of Chagas disease transmission to the human populations, demanding constant monitoring and control actions.

## Figures and Tables

**Table 1:** Localities and years of the left-wing samples of *Triatoma brasiliensis brasiliensis* used for this study. Availability of samples were arranged chronologically, abbreviation are defined as CEIOC: the Entomological Collection of Instituto Oswaldo Cruz and CTIOC: the Collection of Triatomines of Instituto Oswaldo Cruz.

Groups	Year	Collection	State	City	Males	Females	Total
Group 1	1912	CTIOC	Piauí	Floriano	5	1	6
Group 2	1922	CTIOC	Ceará	Quixada	1	0	2
	1929		Rio Grande do Norte	Acary	1	0	
Group 3	1940	CTIOC	Ceará	Russas	12	6	19
	1941		Pernambuco	Mangabeira	1	0	
Group 4	1950	CTIOC	Paraíba	Santa Lúcia	12	7	22
	1955			Santa Lúcia	1	2	
Group 5	1977	CTIOC	Piauí	Brejo Seco	1	3	4
Group 6	1996	CEIOC CTIOC CTIOC	Rio Grande do Norte	Serra Negra do Norte, Caicó	4	5	24
	1998		Piaui	Brejo Seco	2	2	
	1999		Piaui	Brejo Seco	2	9	
Group 7	2002	CEIOC	Paraíba	Livramento, São José da Lagoa da Tapada, São José das Piranhas, São Francisco da Prata	16	15	31
Group 8	2014	CTIOC	Ceará	Santa Quitéria	1	2	3
TOTAL							111

## Data Availability

All insects used in this study are deposited in the entomological collections of Instituto Oswaldo Cruz-Fiocruz.
